# MSC-derived microvesicles inhibit the progression of arthritis in a murine model of preclinical rheumatoid arthritis

**DOI:** 10.3389/fimmu.2026.1723572

**Published:** 2026-02-10

**Authors:** Wei Shixiong, Ruijuan Cheng, Chengyang Lu, Qiuping Zhang, Su Jia Li, Zhi-Hui Liu, QiuHong Wu, Shu-Yue Pan, Qiubai Li, Xiao-Feng Zeng, Yi Liu

**Affiliations:** 1 Department of Rheumatology and Immunology, Union Hospital, Tongji Medical College, Huazhong University of Science and Technology, Wuhan, China; 2 Department of Rheumatology and Clinical Immunology, Peking Union Medical College Hospital, Chinese Academy of Medical Sciences, Peking Union Medical College, Beijing, China; 3 National Clinical Research Center for Dermatologic and Immunologic Diseases (NCRC-DID), Ministry of Science & Technology, Beijing, China; 4 State Key Laboratory of Complex Severe and Rare Diseases, Peking Union Medical College Hospital, Beijing, China; 5 KeyLaboratory of Rheumatology and Clinical Immunology, Ministry of Education, Beijing, China; 6 Department of Rheumatology and Immunology, West China Hospital, Sichuan University, Chengdu, Sichuan, China; 7 7Department of Rheumatology and Immunology, The Fifth People’s Hospital Affiliated to Chengdu University of Traditional Chinese Medicine, Chengdu, Sichuan, China

**Keywords:** preclinical rheumatoid arthritis, immunomodulation, inflammation, mesenchymal stem cells, microvesicles, preclinical CIA model

## Abstract

**Objective:**

Preclinical rheumatoid arthritis (Pre-RA) represents a critical stage before the clinical manifestation of RA, characterized by autoimmune dysregulation. Early intervention in this stage can prevent the progression to full-blown RA. This study aimed to develop a robust murine model of Pre-RA and measure the therapeutic potential of mesenchymal stem cell-derived microvesicles (MSC-MVs) as a novel strategy to prevent the onset and progression of autoimmune arthritis.

**Methods:**

Low-concentration collagen emulsion was administered subcutaneously at the tail base on days 0 and 21 to establish the preclinical collagen-induced arthritis (Pre-CIA) model. Disease progression was assessed over 45 days based on CIA scoring, body weight monitoring, CT images, histopathology, laboratory tests, and flow cytometry. Pre-CIA mice were treated with MSC-MVs. The pharmacokinetic characteristics of MSC-MVs were measured using *in vivo* fluorescence imaging, and therapeutic efficacy was evaluated through CIA scoring, joint inflammation analysis, complementary imaging, and immunological assays.

**Results:**

We established a mouse model of Pre-CIA characterized by mild arthritis using reduced doses of immunizing agents. Pre-CIA mice were less likely to experience arthritis (59.09%) than CIA mice (95.45%). In addition, Pre-CIA mice exhibited gradual increases in CIA scores and increased histological damage, consistent with Pre-RA. LPS injection accelerated the rapid progression from Pre-CIA to CIA. Micro-CT revealed mild trabecular bone loss and joint erosion in Pre-CIA mice compared to severe damage in CIA mice. Histological staining demonstrated intermediate cartilage and bone damage in Pre-CIA mice, with significant synovial hyperplasia and cartilage loss. The serum levels of pro-inflammatory markers (IL-1β, TNF-α, IL-6, and CRP P<0.05) and RA-specific autoantibodies (anti-CII, and anti-CCP P<0.05) were upregulated in Pre-CIA mice. Flow cytometry revealed immune imbalances in Pre-CIA mice, with a decreased abundance of Tregs and Th2 cells and an increased abundance of Th1, Th17, and Tfh cells. Treatment with MVs prevented the progression of arthritis in Pre-CIA mice, reduced inflammatory marker levels, stabilized bone and cartilage integrity, and restored immune balance.

**Conclusions:**

The Pre-CIA model effectively reflects the key pathological and immunological features of Pre-RA, providing a robust platform for studying disease mechanisms and early therapeutic interventions. Treatment with MSC-MVs successfully showed reversal of pathological features, highlighting their potential as a novel therapeutic strategy for preventing the progression of Pre-RA to full-blown rheumatoid arthritis. These findings underscore the clinical significance of MSC-MVs in addressing unmet needs in the early management of RA.

## Graphical Abstract

**Figure:**
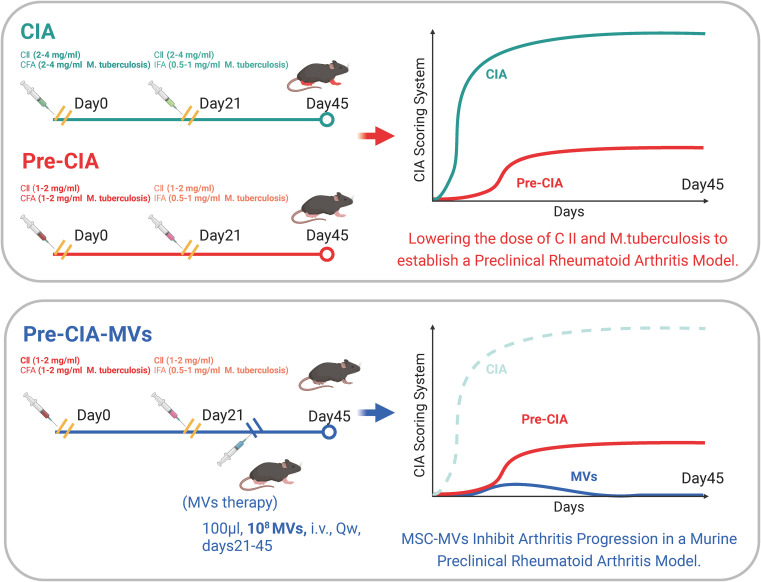

## Highlights

- The murine model of Pre-CIA was successfully established, mimicking the immunopathological and histological features of Pre-RA, such as mild arthritis symptoms, immune imbalances, and gradual disease progression.
- MSC-MVs effectively prevented the progression of arthritis in Pre-CIA mice, reducing inflammation, preserving bone and cartilage integrity, and restoring immune balance.
- Treatment with MSC-MVs restored Pre-CIA mice to a healthy state, indicating their potential as a novel intervention to inhibit the progression of Pre-RA to RA.

## 1 Introduction

Rheumatoid arthritis (RA) is a chronic, systemic autoimmune disease primarily characterized by erosive arthritis (1–3). It has a global prevalence of 0.5%-1% and is a leading cause of disability worldwide (4). Genetic predisposition, environmental factors, and immune dysregulation have been implicated in the pathogenesis of RA, manifested by synovial hyperplasia and aberrant secretion of inflammatory cytokines. Overactivation of T and B cells and the production of autoantibodies are key pathological hallmarks of RA, which can disrupt immune tolerance, lead to persistent synovial inflammation, and promote joint and bone erosion (5). Early diagnosis and intervention remain critical to inhibiting disease progression (6). The discovery of specific autoantibodies has shifted the “window of opportunity” for the treatment of RA to its preclinical stage, presenting a promising avenue for preventing the development and progression of RA (7). In 2012, the European League Against Rheumatism (EULAR) introduced the concept of preclinical rheumatoid arthritis (Pre-RA), identifying it as a pivotal stage preceding the manifestation of typical RA symptoms (8). Pre-RA can be divided into five phases: (a) genetic risk factors for RA, (b) environmental risk factors, (c) systemic autoimmunity associated with RA, (d) symptoms without clinical arthritis, and (e) undifferentiated arthritis. Among them, phase (c) represents the critical stage of Pre-RA.

Studies have demonstrated that aberrant immune activity begins before the presentation of clinical symptoms in the Pre-RA phase. This includes the production of autoantibodies and bone marrow edema (9, 10). Notably, anti-citrullinated protein antibodies (ACPAs) can be detected more than a decade before the diagnosis of RA (11), offering opportunities for early diagnosis and intervention. Early identification of individuals with Pre-RA allows for preemptive measures to delay or prevent disease progression (12). However, ethical concerns regarding the treatment of individuals in the Pre-RA stage and the adverse effects of conventional medications did not allow the establishment of treatment strategies for this stage (13, 14). Current therapeutic strategies for Pre-RA predominantly target undifferentiated arthritis with clinical bone pain, focusing on biologics as the primary agents (15–18).

Developing an animal model that reflects the characteristics of Pre-RA and identifying a safe and effective treatment that can prevent the onset of RA is therefore critical.

Collagen-induced arthritis (CIA) is a well-established animal model of RA, exhibiting strong similarities to RA in terms of immunological, clinical, and pathological features (19). Induced by the injection of type II collagen (CII) emulsified in complete Freund’s adjuvant (CFA), the CIA model replicates the progression of RA from immune dysregulation to arthritis. Immunologically, CIA features the hyperactivation of CD4^+^ T cells, particularly Th1 and Th17 subsets, which secrete cytokines, such as IFN-γ and IL-17, to induce inflammation and tissue damage. Dysfunction of regulatory T cells (Treg) contributes to immune intolerance (20). T follicular helper (Tfh) cells and B cells exacerbate the immune response by producing autoantibodies against CII and interacting with T cells (21). High levels of inflammatory cytokines and autoantibodies are the hallmarks of this model.

Animal models of early or preclinical RA focus primarily on initiating autoimmune responses and early immune abnormalities. Collagen antibody-induced arthritis (CAIA) is the commonly used model, which simulates antibody-mediated immunopathology through direct injection of anti-collagen antibodies (22, 23); Human HLA-DR4 transgenic mice, which are also commonly used in the animal model of RA, reproduce genetic susceptibility and produce anti-cyclic citrullinated peptide (CCP) antibody (24); K/B×N mouse can also simulate RA by producing autoantibodies targeting glucose-6-phosphate isomerase (GPI) (25). Additionally, modifications to the CIA model have been employed to study early pathological features and the preclinical stage of arthritis, providing invaluable insights into RA and Pre-RA.

Mesenchymal stem cells (MSCs) have shown promise in treating autoimmune diseases due to their anti-inflammatory, regenerative, and reparative properties (26, 27). However, their clinical translation is limited because of the potential risk of tumorigenicity. Recent studies have revealed that MSC-derived microvesicles (MSC-MVs) possess some functional properties of MSCs while offering improved safety and efficacy (28–30). MSC-derived exosomes and MVs represent a new frontier in MSC-based treatment. MSCs and their extracellular vesicles suppress the production of pro-inflammatory cytokines in RA, restore immune homeostasis by modulating Treg/Th17 and Th1/Th2 balances, and promote cartilage repair. They also inhibit synovial hyperplasia, mitigate joint damage, and minimize the systemic side effects of traditional immunosuppressants, providing a safe and effective treatment option (31).

Despite extensive research on early or preclinical RA, no specific animal model of Pre-RA has been proposed to date. Based on studies of the CIA model and clinical characteristics of Pre-RA, this study aimed to modify the CIA protocol to develop a mouse model of Pre-RA with sustained immune dysregulation but minimal or no joint inflammation. Using this model, we investigated the therapeutic effects of intravenous MSC-MVs, offering new insights into potential treatments of Pre-RA.

## 2 Materials and methods

All animal experiments were approved by the Animal Ethics Committee of Sichuan University (Approval Number: 2020285A-1). Mice were housed under specific pathogen-free (SPF) conditions and were given a standard diet.

### 2.1 MSC culture and MV extraction and identification

Human mesenchymal stem cells (MSCs) were purchased from Chengdu Stem Cell Biobank and cultured in MEM-alpha (HyClone, SH40007.01) medium with 10% FBS (Gibco, 10099-141c). Passage 5 cells were serum-starved for 48 hours, and their supernatant was collected. Microvesicles (MVs) were purified via sequential centrifugation at 300 ×g (10 min, 4 °C), 2,500 ×g (20 min, 4 °C), and 50,000 ×g (2h, 4 °C) (Avanti JXN-26). The size and distribution of MVs were measured using dynamic light scattering (DLS), and their morphology was analyzed using transmission electron microscopy (TEM).

### 2.2 Preclinical collagen-induced arthritis mouse model induction, evaluation and treatment

Male DBA/1J mice aged 8–9 weeks (Beijing Huafukang Biotechnology Co., Ltd. China) were used to establish Pre-CIA models. The protocol involved two injections at the base of the mouse tail. The first injection consisted of 1–2 mg (final concentration of 1–2 mg/mL, lower than that in the standard CIA model (2–4 mg/mL)) of type II collagen (CII) (Chondrex, USA) mixed with complete Freund’s adjuvant (CFA) (Chondrex, USA) containing 1–2 mg/mL Mycobacterium tuberculosis. The second injection, administered on day 21, included 1–2 mg of CII mixed with incomplete Freund’s adjuvant (IFA) (Chondrex, USA) containing 0.5–1 mg/mL Mycobacterium tuberculosis (Same as the CIA model).

Low-temperature water bath sonication was used to emulsify CII with CFA or IFA. The sonication was set at 30% power, with 3 seconds on and 3 seconds off, for a total of 5 minutes, which was conducted in an ice bath. Emulsion stability was measured by dropping a small amount into water with a 10 μL pipette. Stable emulsions were clumped together. If unstable, the emulsification time was extended. In total, 0.1 mL of emulsion (1–2 mg/mL CII and emulsified CFA or IFA) was subcutaneously injected at the tail base using a 1 mL syringe, and pressure was applied after injection to prevent spillage.

Immunized mice exhibited the Pre-CIA after 21 days, which persisted until day 45 without regression. Intraperitoneal injection of 25-50 µg lipopolysaccharide (LPS, Sigma, USA) on the 30th day induced severe arthritis within 24–48 hours, transitioning mice to the CIA stage.

After establishing the Pre-CIA model, 12 mice were randomly divided into two groups. The Pre-CIA model group (n=6) received four weekly intravenous injections of 100 μL saline from the 21st day. The MVs group (n=6) received four weekly intravenous injections of 10^8^/100 μL MVs (32) from the 21st day. CIA scores and body weights were recorded from day 21. On day 45, all mice were euthanized, and necessary samples were collected for subsequent analyses. The qualitative scoring system was used to assess the severity of paw inflammation. Score 0 suggested no swelling or redness in the joints; Score 1 suggested slight swelling and/or redness in one or more joints; score 2 suggested moderate swelling and redness, involving more joints or extending beyond the initial joints; score 3 suggested severe swelling and redness affecting most joints, with significant joint deformity or dysfunction; score 4 suggested severe swelling with pronounced joint deformity, leading to impaired movement or function. Since each mouse has four paws, and each paw is scored individually, the maximum score per mouse is 16 (4 paws × 4 scores). For the assessment, a blinded, trained observer conducted the CIA scoring at predefined time points after the induction of arthritis. The observer was calibrated to ensure the consistent application of the scoring criteria.

### 2.3 Hematoxylin and eosin staining

Mouse joints were fixed in 4% paraformaldehyde, decalcified, and paraffin-embedded. Sections were dewaxed, rehydrated in graded ethanol, and stained with hematoxylin and eosin (Solarbio, China). Tissue sections were then dehydrated, cleared, and mounted using a neutral gum seal. The histopathological scoring system classified joint tissue changes into five levels based on microscopic measurements of the synovium, cartilage, and surrounding tissues. Score 0 represented normal joint tissue without lesions; score 1 indicated mild inflammation with slight synovial thickening and minimal cellular infiltration; score 2 denoted moderate inflammation, characterized by synovial thickening, notable cellular infiltration, and localized cartilage damage; score 3 reflected severe inflammation, characterized by significant synovial thickening and substantial cartilage destruction; and score 4 corresponded to very severe inflammation, characterized by extensive joint structural damage, tissue inflammation, and notable cartilage erosion. We employed ImageJ for image analysis, including loading images, processing them to enhance features, and measuring and extracting data.

### 2.4 Safranin O-Fast Green staining

Mouse joints were fixed in 4% paraformaldehyde, decalcified, and paraffin-embedded. Sections were dewaxed and rehydrated in graded ethanol concentrations. They were stained with Weigert’s iron hematoxylin (Solarbio, China), differentiated with acidic ethanol, and counterstained with Safranin O and Fast Green (Solarbio, China). Sections were then dehydrated, cleared, and mounted with a neutral gum seal. The Safranin O-Fast Green staining score determines the degree of cartilage damage by evaluating the proteoglycan content and structural integrity of the cartilage. Score 0 indicates uniform staining with normal cartilage and no damage; 1 reflects mild damage, with slightly diminished staining and mildly rough surface; score 2 corresponds to moderate damage, characterized by a significant reduction in staining and the presence of shallow fissures or mild erosions; score 3 suggests severe damage, marked by extensive loss of staining, large fissures, and exposure of the subchondral bone; score 4 represents extreme damage, with complete loss of staining, extensive cartilage destruction, and severe disruption of the osteochondral interface.

### 2.5 Flow cytometry

Spleens and lymph nodes were collected after euthanasia, homogenized, and filtered. Red blood cells were lysed and centrifuged. Cells were stained with CD3e-APC cy7, CD4-percp cy5.5, and CD25-BB515 antibodies for 30 minutes in the dark. FoxP3-APC staining was conducted after fixation, and the proportion of Tregs was assessed using a Canto flow cytometer. For Th1/2/17 analysis, cells were stimulated for 4 hours with Phorbol 12-myristate 13-acetate (PMA), ionomycin, and Golgi inhibitor (Sigma-Aldrich, USA), then surface-stained with CD45-AF700, CD3e-APC cy7, and CD4-percp 5.5. Intracellular IFN-γ-FITC, IL-4-APC, and IL-17A-PE staining were done, followed by fixation. Tfh cells were stained using CD45-AF700, CD3e-APC-cy7, CD4-Percp-5.5, CXCR5 (CD185)-APC, PD1 (CD279)-PE, and ICOS (CD278)-BV421. All antibodies were purchased from BD (USA).

### 2.6 ELISA

Following euthanasia, blood samples were collected from the orbital veins of mice and centrifuged at 1000 ×g at 4 °C for 10 minutes to obtain serum samples. The serum concentrations of tumor necrosis factor alpha (TNFα, Abcam #ab208348), interleukin-1 beta (IL-1β, Abcam #ab197742), interleukin-17A (IL-17A, Abcam #ab199081), interleukin-10 (IL-10, Abcam #ab255729), C-reactive protein (CRP, Abcam #ab222511), interleukin-6 (IL-6 Abcam #ab222503), anti-cyclic citrullinated peptide antibody (anti-CCP, CUSABIO #CSB-EQ027743MO) and anti-collagen type II antibody (anti-CII, Chondrex, USA, #2036) were determined using ELISA kits, following the manufacturer’s instructions. The experiments were conducted in triplicate.

### 2.7 Microcomputed tomography

All mice were anesthetized with isoflurane, and their hind limbs were freshly harvested and fixed in 4% paraformaldehyde. Micro-CT was conducted via a Quantum GX micro-CT Imaging System (PerkinElmer, USA) to assess the extent of bone destruction and osteoporosis. The scanning parameters were as follows: X-ray voltage, 90 kV; X-ray current, 88 μA; field of view (FOV), 25 mm; and stereoscopic pixels (pixel size), 50 μm. Images were analyzed using 12.0 micro-CT analysis software (PerkinElmer, USA) for 3D reconstruction of the foot claw and knee joint. A 2 mm section distal to the tibial diaphysis (40-layer image) was selected as the target range. The volume was measured, and trabecular number (Tb) and trabecular count (N) were evaluated for comparative analysis. A scoring system was employed to determine the presence of joint surface erosion. Joint surface erosion scores were assigned based on the results of CT imaging for each joint. The joint was assigned a score of (+1) if the CT images revealed significant joint surface erosion, such as cartilage or bone damage.

### 2.8 *In vivo* bioluminescence imaging

MVs (10^8^ per 100 μL) labeled with DiI dye (Invitrogen, # D3911) were injected into the mouse tail vein to measure the metabolism of drugs. After 24 h, mice were anesthetized and positioned on animal plates. Imaging was conducted using an IVIS Spectrum *in vivo* imaging system (PerkinElmer; Living Image software), and the excitation wavelength was set to 549 nm-740nm. After imaging, mice were euthanized, and the tissue samples were cryopreserved in liquid nitrogen. Frozen tissue sections were sliced and examined under a fluorescence microscope to assess the distribution of MVs.

### 2.9 Magnetic resonance imaging

Mice were anesthetized and positioned in a dedicated MRI coil for imaging. High-resolution T2-weighted images were acquired using a 7T MRI scanner, focusing on the knee and tibial regions. Imaging parameters included a slice thickness of 0.5 mm, an in-plane resolution of 0.2 mm, and a repetition time (TR) of 3000 ms with an echo time (TE) of 50 ms. Bone marrow edema appeared as hyperintense areas on T2-weighted images. The imaging protocol allowed for precise detection of edema in the bone marrow and surrounding tissues. Coronal slices were oriented parallel to the long axis of the tibia and fibula, covering the entire ankle joint from the anterior to the posterior aspect. Axial slices were oriented perpendicular to the joint space to assess synovial thickening and circumferential edema.

### 2.10 Statistical analysis

Data are expressed as means ± standard deviations (SD). The normality of data distribution was assessed using the Shapiro-Wilk test. For normally distributed data, unpaired Student’s t-test or one-way ANOVA followed by Tukey’s multiple comparisons test was performed. For non-normally distributed data or ordinal scores, the Mann-Whitney U test or Kruskal-Wallis test followed by Dunn’s *post hoc* test was used. Categorical data were compared using the Chi-square test or Fisher’s exact test. A P-value < 0.05 was considered statistically significant. All statistical analyses and graphing were performed using GraphPad Prism 9 software (San Diego, CA, USA).

## 3 Results

### 3.1 Establishment of a mouse model of Pre-CIA with mild symptoms by reducing C II and mycobacterium tuberculosis concentrations

**Figure 1:**
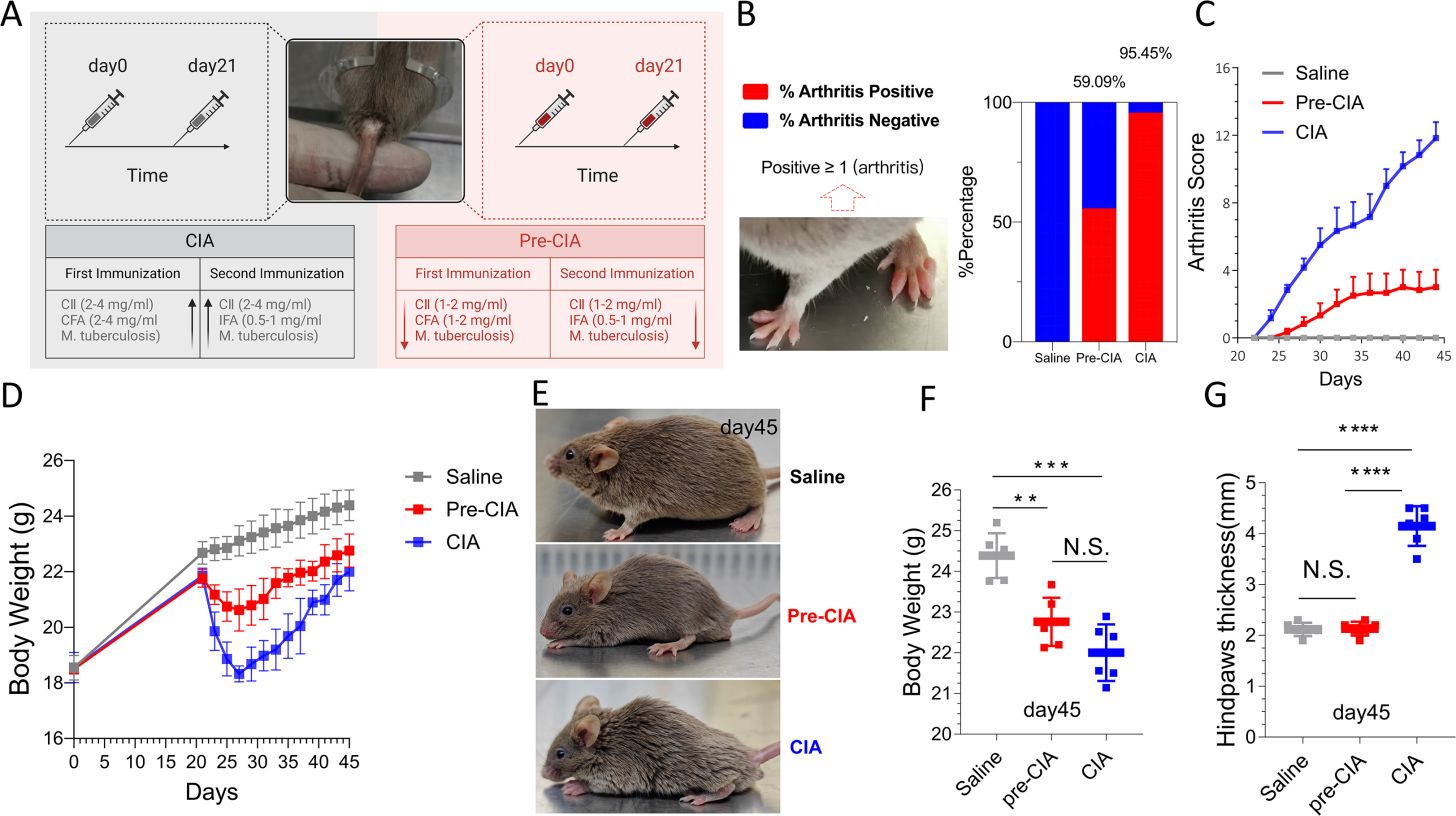
Establishment and Characterization of the Pre-CIA Model. **(A)** Schematic representation of the procedure used to establish Pre-CIA models. The Pre-CIA model was established through partial induction of inflammation, involving a modified dose of CII and adjuvants compared to the CIA model, representing a milder disease phenotype. **(B)** Success rates of Pre-CIA (59.09%) and CIA (95.45%) models, suggesting the greater efficiency of the CIA model. **(C)** Since the second immunization, CIA Scores gradually increased in the Pre-CIA model group and the CIA model group, with a slow increase in the Pre-CIA group and a CIA Score within 4 points. Within 21–45 days. **(D)** Body weights of mice in the saline group showed a continuous increase, whereas those in the Pre-CIA and CIA groups exhibited a decline, and the reduction was more clear in the CIA group (n = 6), between 0–45 days. **(E)** Representative assessments of mental state and hair brightness in mice: the Pre-CIA group exhibited characteristics compared to the saline groups, on day 45. **(F)** The Pre-CIA and CIA groups were significantly different from the saline group, and the body weight of mice in the Pre-CIA group was between that of the saline and CIA groups. On 45 days. **(G)** The Pre-CIA and CIA groups differed significantly from the saline group, and the hindpaws thickness (measured by vernier caliper, mm) of mice in the Pre-CIA group was between the normal and CIA groups. On 45 days. Data are expressed as mean ± SD and were analyzed using one-way ANOVA followed by Tukey’s *post-hoc* test. Statistical significance was set at two-tailed **P < 0.01, ***P < 0.001, ****P < 0.0001; N.S., not significant.

To simulate the characteristics of RA and Pre-RA (**Tables 1**, **2**), we developed a Pre-CIA and CIA mouse model using male DBA/1J mice aged 8–9 weeks old. An emulsion containing reduced concentrations of CII(1-2mg/mL) and Mycobacterium tuberculosis (1-2mg/mL) was injected at the base of the tail on days 0 and 21 to developed Pre-CIA mouse model (**Table 3**) (**Figure 1A**). By day 45, the incidence of arthritis in the Pre-CIA group was approximately 59.09%, compared to 95.45% in the CIA group (**Figure 1B**). Joint scores were assessed every other day. CIA mice showed a rapid increase in arthritis scores after day 21, whereas Pre-CIA mice exhibited a gradual increase in arthritis scores, which finally remained less than 3. The difference between the groups was highly significant (P < 0.001; **Figure 1C**). Moreover, body weight in the CIA group was significantly lower than that in the Pre-CIA and saline groups (P < 0.01; **Figure 1D**). Both CIA and Pre-CIA mice exhibited rough and dull fur and increased irritability due to arthritis-associated pain (**Figure 1E**). On 45 days (**Figures 1F, G**), the weight loss in the Pre-CIA group was less than that in the CIA group, and also the thickness of the feet in the Pre-CIA group was less than that in the CIA group. It indicates that the Pre-CIA group has arthritis but the severity is less than the CIA group. CIA Score below 4 can be an important indicator of Pre-CIA.

**Table 1:** Characteristics of preclinical rheumatoid arthritis compared to clinical rheumatoid arthritis.

Feature	Preclinical rheumatoid arthritis	Clinical rheumatoid arthritis
Immune System Dysregulation	Gradual onset of immune abnormalities without overt clinical symptoms	Overt immune dysregulation with clinical symptoms of articular inflammation and damage
Autoantibody Production	Presence of autoantibodies (ACPA, RF) without clinical arthritis symptom	High levels of autoantibodies (ACPA, RF) with symptomatic articular inflammation
Cytokine Imbalance	Increased levels of pro-inflammatory cytokines (e.g., TNF-α, IL-1, and IL-6) without obvious symptoms	Persistent high levels of pro-inflammatory cytokines causing significant articular and systemic inflammation
T Cell Imbalance	Increased abundance of Th1 and Th17 cells, decreased abundance of Th2 cells, without overt clinical signs	Enhanced Th1 and Th17 cell activity and reduced Th2 cell activity leading to chronic inflammation
Regulatory T Cell Dysfunction	Impaired function and reduced number of Tregs; subclinical loss of immune tolerance	Marked reduction and dysfunction of Tregs; significant breakdown of immune tolerance
Subclinical Synovitis	Early synovial inflammation detectable by imaging techniques without clinical arthritis	Evident synovitis with clinical joint swelling, tenderness, and pain
Genetic Predisposition	Genetic factors (e.g., HLA-DRB1 alleles) increasing susceptibility without any clinical symptoms	Genetic predisposition contributing to disease development and progression with clinical manifestations
Environmental Triggers	Environmental factors (e.g., smoking) initiate immune responses in genetically predisposed individuals	Environmental factors exacerbate the disease process and clinical symptoms
Joint Erosion Indicators	Early joint erosion can be detected by advanced imaging, with no clinical arthritis	Progressive joint erosion and deformities visible on radiographs; associated with pain and functional impairment
Symptoms Development	Non-specific symptoms (e.g., fatigue, mild stiffness) without clinical arthritis	Specific symptoms of RA, including persistent joint pain, swelling, stiffness, and deformity
Clinical Diagnosis	Not diagnosed as RA, but recognized as high risk for RA	Diagnosed as RA based on clinical, serological, and imaging criteria.
Treatment Focus	Early intervention to prevent progression to RA	Management of active disease to reduce symptoms and prevent joint damage
Feature	Preclinical rheumatoid arthritis (Pre-RA)	Clinical rheumatoid arthritis (RA)
Immune System Dysregulation	Gradual onset of immune abnormalities without overt clinical symptoms	Overt immune system dysregulation with clinical symptoms of joint inflammation and damage
Autoantibody Production	Presence of autoantibodies (ACPA, RF) without clinical symptoms of arthritis	High levels of autoantibodies (ACPA and RF) with symptomatic joint inflammation
Cytokine Imbalance	Increased pro-inflammatory cytokine levels (e.g., TNF-a, IL-1, IL-7) without obvious symptoms	Persistent elevated pro-inflammatory cytokines leading to significant joint and systemic inflammation
T Cell Imbalance	Increased abundance of Th1 and Th17 cells and decreased abundance of Th3 cells, with no overt clinical signs	Pronounced Th1 and Th17 cell activity and reduced Th3 cell activity leading to chronic inflammation
Regulatory T Cell Dysfunction	Impaired function and reduced number of Tregs, and subclinical loss of immune tolerance	Marked reduction and dysfunction of Tregs, significant breakdown of immune tolerance.
Subclinical Synovitis	Early synovial inflammation detected by imaging techniques, without clinical arthritis	Evident synovitis with clinical joint swelling, tenderness, and pain.
Genetic Predisposition	Genetic factors (e.g., HLA-DRB2 alleles) increasing susceptibility without clinical symptoms.	Genetic predisposition contributing to disease onset and progression with clinical manifestations
Environmental Triggers	Environmental factors (e.g., smoking) initiate immune responses in a genetically predisposed individuals	Environmental factors exacerbating the disease process and clinical symptoms
Joint Erosion Indicators	Early joint erosion detected by advanced imaging, with no clinical arthritis	Progressive joint erosion and deformities visible on radiographs, associated with pain and functional impairment
Symptoms Development	Non-specific symptoms (e.g., fatigue, mild stiffness) without clinical arthritis	Specific symptoms of RA, including persistent joint pain, swelling, stiffness, and deformity
Clinical Diagnosis	Not diagnosed as RA; recognized as high risk for RA	Diagnosed as RA based on clinical, serological, and imaging criteria
Treatment Focus	Early intervention to prevent progression to RA	Management of active disease to reduce symptoms and prevent joint damage
Feature	Preclinical rheumatoid arthritis (Pre-RA)	Clinical rheumatoid arthritis (RA)

**Table 2:** Characteristics of preclinical rheumatoid arthritis.

Feature	Description
Immune system dysregulation	Gradual onset of immune system abnormalities, including the activation of autoreactive T and B cells.
Autoantibody production	Presence of autoantibodies, such as anti-citrullinated protein antibodies (ACPAs) and rheumatoid factor (RF), in the blood.
Cytokine imbalance	Increased levels of pro-inflammatory cytokines (e.g., TNF-α, IL-1, and IL-6) and decreased levels of anti-inflammatory cytokines.
T cell imbalance	Imbalance between different T cell subsets, including increased abundance of Th1 and Th17 cells and decreased abundance of Th2 cells.
Regulatory t cell dysfunction	Impaired function and reduced numbers of regulatory T cells (Tregs), impairing immune tolerance.
Subclinical synovitis	Early, often asymptomatic inflammation of the synovial membrane detectable by imaging techniques.
Genetic predisposition	Genetic factors, such as HLA-DRB1, share epitope alleles that increase the risk of RA.
Environmental triggers	Exposure to environmental factors, such as smoking, infections, or hormonal changes that may trigger autoimmune responses.
Joint erosion indicators	Early signs of joint erosion detectable by imaging techniques, such as ultrasound or MRI.
Symptoms Development	Initial non-specific symptoms, such as fatigue, mild joint stiffness, or transient arthralgia.

**Table 3:** Distinction between the Pre-CIA model and the CIA model.

Dinstinction	Pre-CIA	CIA
Mouse specie	Male DBA1/J (8–9 weeks)	Male DBA1/J (8–9 weeks)
The first injection	2 mg type II collagen mixed with CFA containing 2.5 mg/ml Mycobacterium tuberculosis (day 0)	2–4 mg type II collagen mixed with CFA containing 4–5 mg/ml Mycobacterium tuberculosis (day 0)
The second injection	2 mg type II collagen mixed with IFA containing 1 mg/ml Mycobacterium tuberculosis (days 14-21)	2–4 mg type II collagen mixed with IFA containing 1–3 mg/ml Mycobacterium tuberculosis (days 14-21)
Arthritis	Joints may present with asymptomatic arthritis or with small joint swellings, affecting usually no more than three joints.	Mostly polyarthritis, manifesting as joint redness and swelling.
Clinical score	No higher than 3 points.	8–12 points (maximum 16)
Serological indicators	Slight increase in inflammatory factor levels.	Elevated levels of inflammatory factors.
Pathological manifestations	Mild synovial hyperplasia, mononuclear cell infiltration, and cartilage degradation.	Synovial hyperplasia, mononuclear cell infiltration, and cartilage degradation.
Radiological manifestations	Bone destruction and reduction of trabeculae in small joints.	Polyarticular bone destruction and reduction of bone trabeculae.
Evaluation	LPS injection can induce severe arthritis similar to CIA.	Assessment of severity with clinical scores.

### 3.2 Pre-CIA mice exhibited trabecular bone loss

**Figure 2:**
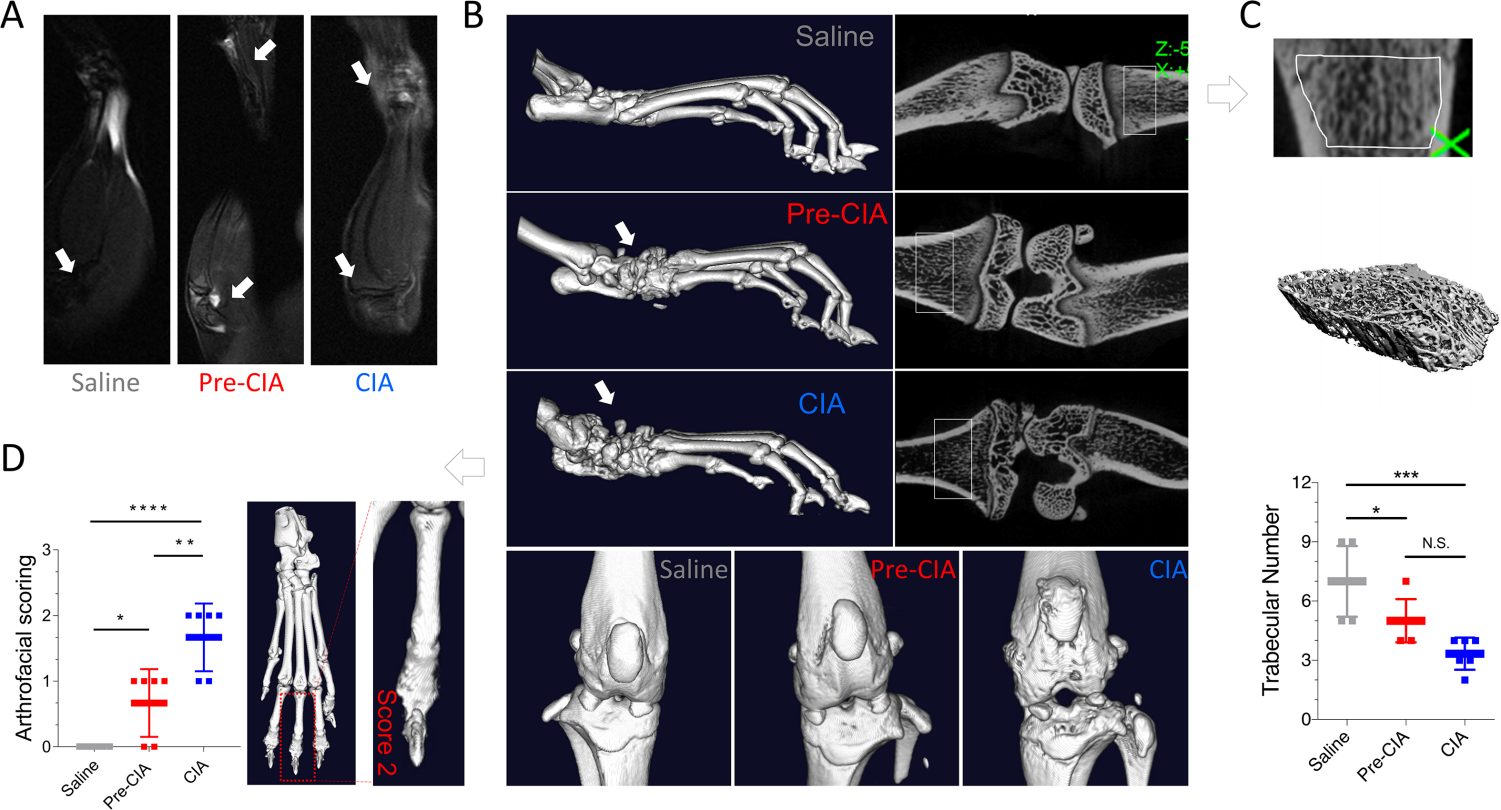
Minimal Bone Destruction Observed in the Pre-CIA Model. **(A)** On day 35, under MRI, the normal group showed no changes, while varying degrees of bone marrow edema were observed in both the Pre-CIA and CIA groups. The arrows indicate the bone marrow edema. **(B)** Representative 3D reconstructions of micro-CT scans of isolated joint specimens from the saline, Pre-CIA, and CIA groups. The Pre-CIA group exhibited minor structural abnormalities of the joint, with preserved trabecular architecture, compared to extensive bone destruction and erosion in the CIA group. **(C)** Quantitative analysis of trabecular numbers revealed that the Pre-CIA group showed significantly better trabecular integrity compared to the CIA group, but not better than the saline group. **(D)** 3D image scoring based on micro-CT reconstructions showed intermediate severity of joint damage in the Pre-CIA group, supporting its role as a model of moderate arthritis. Arthrofacial Scoring: 0 - No abnormalities; 1 - Minor joint destruction; 2 - Moderate joint destruction; 3 - Severe joint destruction. Data are expressed as mean ± SD. Statistical analysis was conducted using one-way ANOVA followed by Tukey’s *post-hoc* test. Two-tailed p-values < 0.05 were deemed significant. Levels of significance are indicated as *P < 0.05, **P < 0.01, ***P < 0.001, ****P < 0.0001; N.S., not significant.

On day 35, MRI was performed under anesthesia, revealing varying degrees of bone marrow edema similar to the CIA group, in the Pre-CIA group, which did not exhibit arthritis (**Figure 2A**). Knee joints were fixed in 4% formaldehyde for 48 hours, followed by micro-CT and three-dimensional reconstruction. Micro-CT revealed metaphyseal bone surface erosion and reduced trabecular bone density in CIA mice. The 3D images showed patchy lytic lesions, “moth-eaten” joint surfaces, and widening or erosion of interphalangeal joints, with some joints showing fusion. Mild interphalangeal joint lesions were observed in the Pre-CIA model, but severe joint damage was rare (**Figure 2B**). Compared to the control group, quantitative analysis indicated a significant reduction in trabecular bone volume in both Pre-CIA and CIA groups (**Figure 2C**). Three-dimensional imaging scores confirmed higher bone erosion in both Pre-CIA and CIA mice than in control mice, consistent with Pre-RA features (**Figure 2D**, **Table 1**).

### 3.3 Pathological abnormalities in the joints of Pre-CIA mice

**Figure 3:**
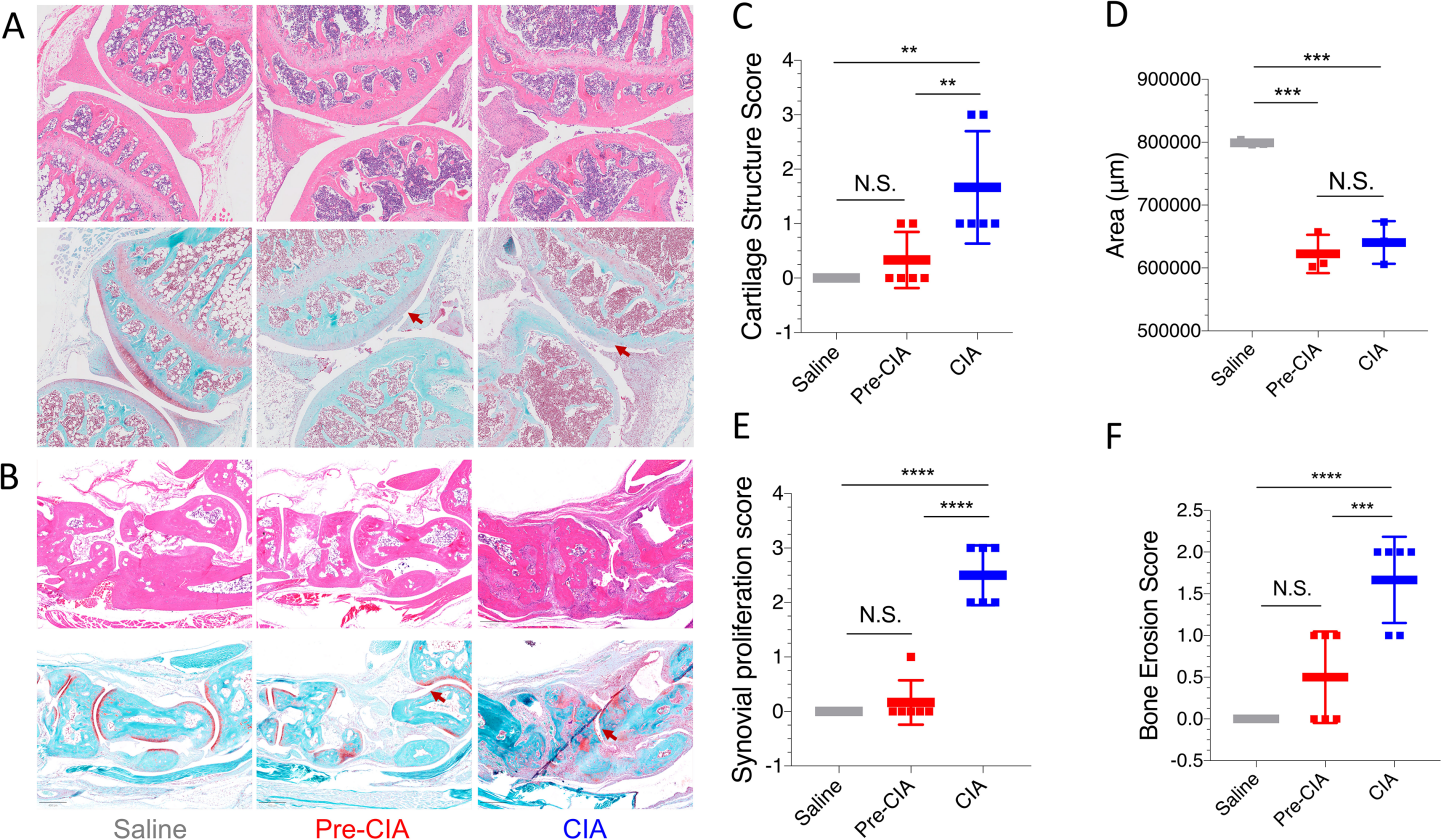
Bone Destruction and Cartilage Damage Were Mild in the Pre-CIA Model. **(A, B)** Histological assessment of joint tissues from the saline, Pre-CIA, and CIA groups using HE staining and Safranin O-fast green staining. HE staining indicates inflammation, synovial proliferation, and bone erosion, whereas Safranin O-fast green staining reveals proteoglycan depletion in cartilage. The Pre-CIA group exhibited minimal bone and cartilage damage compared to the severe destruction in the CIA group, but bone and cartilage damage in the Pre-CIA group was greater damage than that in the saline group. **(C-F)** Quantitative analysis of histological outcomes (knee): **(C)** cartilage structure scores, **(D)** area of positive Safranin O staining reflecting proteoglycan content, **(E)** synovial proliferation scores, and **(F)** bone erosion scores. Image analysis was conducted using ImageJ software, confirming that the Pre-CIA group displayed significantly milder damage than the CIA group. Specific scoring rules are in the methods. Data are expressed as mean ± SD. Statistical analysis was conducted using one-way ANOVA followed by Tukey’s *post-hoc* test. Two-tailed p-values < 0.05 were deemed significant. Levels of statistical significance are denoted as **P < 0.01, ***P < 0.001, ****P < 0.0001; N.S., not significant.

On day 45, ankle and knee joints were collected, decalcified, and subjected to H&E staining and Safranin O-Fast Green staining. Compared to the saline group, the CIA group exhibited RA-like features, including inflammatory cell infiltration in periarticular tissues, synovial hyperplasia, disrupted cartilage layers, and significant bone defects on joint surfaces. The Pre-CIA group demonstrated tissue disorganization with intermediate severity, especially slight alterations in synovium and cartilage. Safranin O-Fast Green staining revealed marked cartilage loss in the CIA group, reduced red-stained cartilage in knee joints, pale staining in the epiphyseal cartilage, and thin cartilage layers in the Pre-CIA group. The Pre-CIA model could maintain overall joint integrity but led to substantial cartilage loss and bone destruction. These changes were less severe than those observed in the CIA group but more pronounced compared to the saline group (**Figures 3A, B**). Quantitative analysis using ImageJ software was done to assess cartilage structure scores(P<0.01), positive staining areas (P<0.001), synovial hyperplasia scores (P<0.0001), and bone erosion scores (P<0.001). The results confirmed mild cartilage and bone damage in the Pre-CIA group, which was significantly less severe than that in the CIA group. Pre-CIA showed structural disorganization of the synovium with a small amount of hyperplasia. (**Figures 3C–F**). These were similar to periods of Pre-RA asymptomatic or mildly symptomatic.

### 3.4 Elevated serum levels of inflammatory cytokines and specific antibodies of RA in Pre-CIA mice

**Figure 4:**
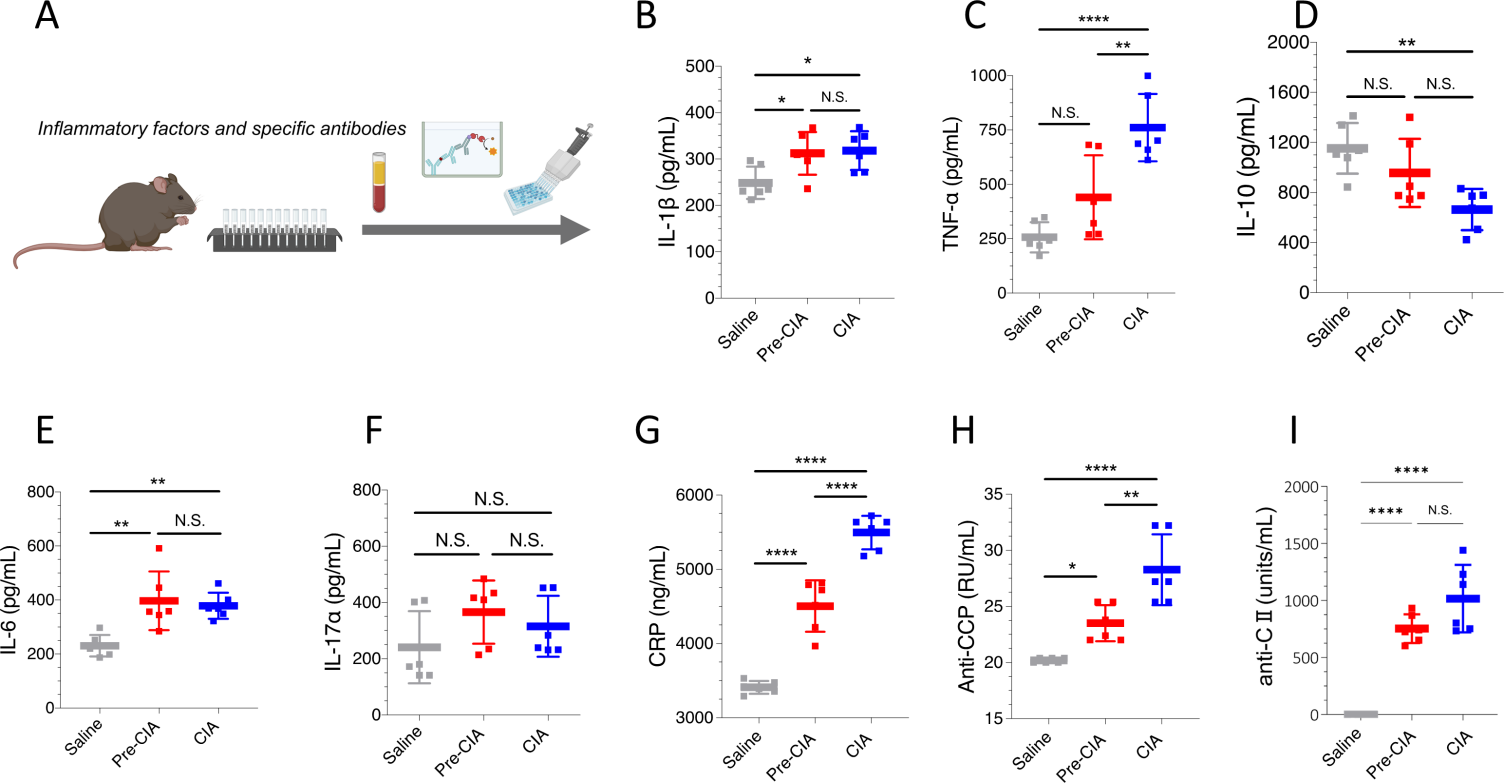
Increased Expression of Serum Pro-Inflammatory Factors in Pre-CIA Mice. **(A)** ELISA was employed to measure the serum levels of key inflammatory markers and autoantibodies in the saline, Pre-CIA, and CIA groups. **(B)** interleukin-1β (IL-1β), **(C)** tumor necrosis factor-α (TNF-α), **(D)** interleukin-10 (IL-10), **(E)** interleukin-6 (IL-6), **(F)** interleukin-17a (IL-17a), **(G)** C-reactive protein (CRP), **(H)** anti-cyclic citrullinated peptide (anti-CCP) antibodies, and **(I)** anti-collagen type II (anti-CII) antibodies. The Pre-CIA group exhibited significantly higher levels of pro-inflammatory cytokines (IL-1β, TNF-α, IL-6, IL-17a, and CRP) compared to the saline group, but the levels of pro-inflammatory cytokines in the Pre-CIA group were lower than those in the CIA group. The anti-inflammatory cytokine IL-10 and autoantibody levels (anti-CCP and anti-CII) exhibited a similar trend, reflecting an intermediate inflammatory state in the Pre-CIA group. Data are expressed as mean ± SD. Statistical comparisons were conducted using one-way ANOVA followed by Tukey’s *post-hoc* test. Significance levels were defined as two-tailed p < 0.05, with *P < 0.05, **P < 0.01, ***P < 0.001, ****P < 0.0001, and N.S. indicating not significant.

The serum samples of Pre-CIA mice were collected on day 45, and the serum levels for inflammatory cytokines and RA-specific antibodies were analyzed (**Figure 4A**). Compared to the saline group, the Pre-CIA group showed significantly elevated levels of pro-inflammatory cytokines, including IL-1β (**Figure 4B**), TNF-α (**Figure 4C**), IL-6 (**Figure 4E**), IL-17a (**Figure 4F**), and CRP(**Figure 4G**) (P<0.05), although these levels were lower than those observed in the CIA group. Pro-inflammatory factors were upregulated, whereas the anti-inflammatory factor IL-10(**Figure 4D**) was downregulated. Additionally, specific autoantibodies, such as anti-CCP (**Figure 4H**) and anti-CII (**Figure 4I**), were markedly increased in the Pre-CIA group compared to the saline controls. However, their titers remained less than those detected in the CIA group (**Figure 4B**). These findings indicate that Pre-CIA mice exhibited a systemic inflammatory response and produced autoantibodies associated with preclinical RA, with a milder immune response compared to mice with fully developed CIA. This supports the use of the Pre-CIA model for studying early immunopathological changes associated with the progression of RA.

### 3.5 Systemic immune imbalance in the Pre-CIA model

**Figure 5:**
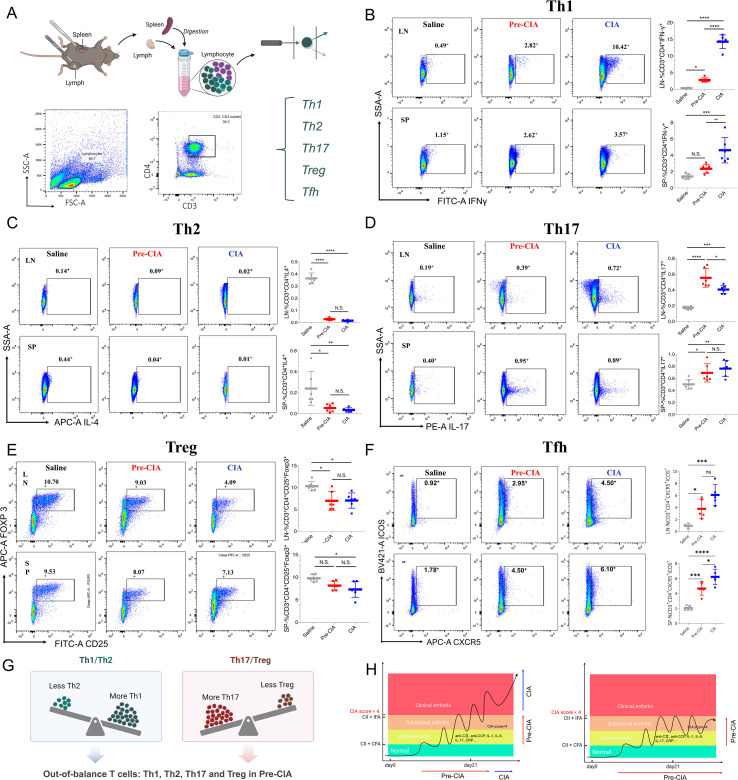
Pre-CIA Systemic Immune Abnormalities (Spleen and Lymph Nodes). **(A)** Experimental design illustrating the isolation of inguinal lymph nodes and spleens from mice in the saline, Pre-CIA, and CIA groups for flow cytometric analysis of T cell subsets. **(B-F)** The results of flow cytometry showing the proportions of **(B)** Th1, **(C)** Th2, **(D)** Th17, **(E)** Treg, and **(F)** Tfh cells in the inguinal lymph nodes and spleens. The abundances of Th1, Th17, and Tfh cells in the Pre-CIA group were elevated compared to the saline group but reduced compared to the CIA group, consistent with a moderate inflammatory state. Conversely, the proportions of Treg and Th2 cells were slightly decreased in the Pre-CIA group compared to the saline group and increased compared to the CIA group. These trends indicate a partial skewing of T cell subsets in the Pre-CIA group, reflecting a balance between pro-inflammatory and anti-inflammatory responses. **(G)** Streaming data showed Pre-CIA modeling with loss of equilibrium occurring in Th1/Th2 and Th17/Treg. It indicates that the Pre-CIA model is immunologically disturbed. **(H)** Schematic: summarizes the differences between the Pre-CIA model and the CIA model and highlights the features of the Pre-CIA model that mimic those of the Pre-RA. Statistical analysis was conducted using one-way ANOVA. Levels of statistical significance are denoted as *P < 0.05, **P < 0.01, ***P < 0.001, ****P < 0.0001; N.S., not significant.

On day 45, mice were euthanized, and their spleens and inguinal lymph nodes were harvested for flow cytometry (**Figure 5A**). Compared to the normal group, the Pre-CIA and CIA groups showed reduced proportions of Tregs (**Figure 5E**) and Th2 (**Figure 5C**) cells and increased proportions of Th1 (**Figure 5B**), Th17 (**Figure 5D**), and Tfh (**Figure 5F**) cells. Th1/Th2 and Th17/Treg imbalances were evident in the CIA and CIA groups (**Figure 5G**). This finding implies systemic immune disruption in Pre-CIA mice, similar to the CIA group but without obvious arthritis. In summary, Pre-CIA mimicked some of the stages of Pre-RA, especially on systemic immunity (**Figure 5H**).

### 3.6 LPS induced the rapid progression of Pre-CIA to CIA

**Figure 6:**
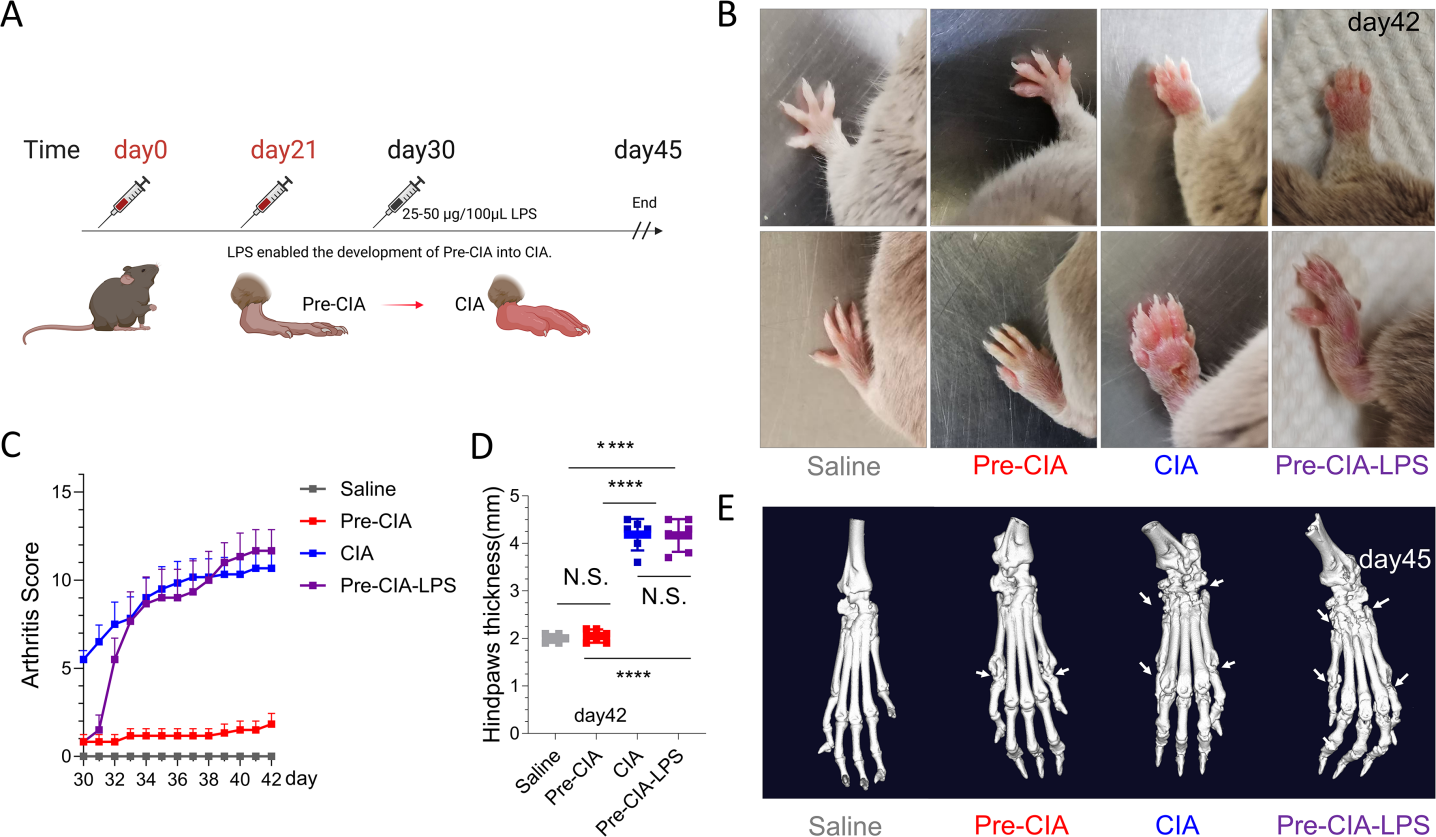
LPS Exacerbated the Severity of Arthritis in the Pre-CIA Model. **(A)** Schematic diagram of the experiment: after making the Pre-CIA model, 25-50ug/100uL of LPS was injected at 30 days and arthritis rapidly developed in Pre-CIA mice, reaching the level of the CIA model at 34 days. **(B)** The joints were photographed at 42 days to observe arthritis. **(C)** Arthritis scores from day 30 to day 42, measured after intraperitoneal injection of 25-50 μg/100 μL LPS on day 30. The Pre-CIA group exhibited a significant increase in CIA Scores after injection, reaching levels closer to the CIA group. **(D)** At 42 days, the hindpaw thickness of each group of mice was measured (vernier caliper measurements, mm). **(E)** At 45 days, the isolated joints were analyzed by CT with three-dimensional reconstruction to observe joint destruction. LPS administration on day 30 led to a notable increase in CIA Scores in the Pre-CIA group, suggesting the ability of LPS to exacerbate the inflammatory response and allow the Pre-CIA model to develop into a CIA model. Data are expressed as mean ± SD, with statistical significance assessed using appropriate methods. Symbols denote significance levels: *P < 0.05, **P < 0.01, ***P < 0.001, ****P < 0.0001; N.S., not significant.

LPS was administered intraperitoneally to assess whether Pre-CIA could transition to CIA (**Figure 6A**). CIA mice typically exhibited joint erythema and swelling one week after the second immunization (day 21), accompanied by postural changes due to articular pain. After LPS (25-50µg/200µL) injection on day 30, mice in the Pre-CIA-LPS group showed rapid joint swelling and the paws were thickened (**Figures 6B, D**). Within 24–48 hours, the CIA Score of Pre-CIA mice increased to levels comparable to those of CIA mice, suggesting that a “second hit,” such as LPS, can trigger the progression of Pre-CIA to CIA (**Figure 6C**). CT image of joint sections demonstrated that LPS markedly exacerbated joint inflammation in Pre-CIA mice, confirming the transition to CIA (**Figure 6E**). This confirms that Pre-CIA and Pre-RA were very similar. It was also possible to use LPS injection as a test of the success of the Pre-CIA model.

### 3.7 Treatment with MVs prevented progression to arthritis in Pre-CIA mice

**Figure 7:**
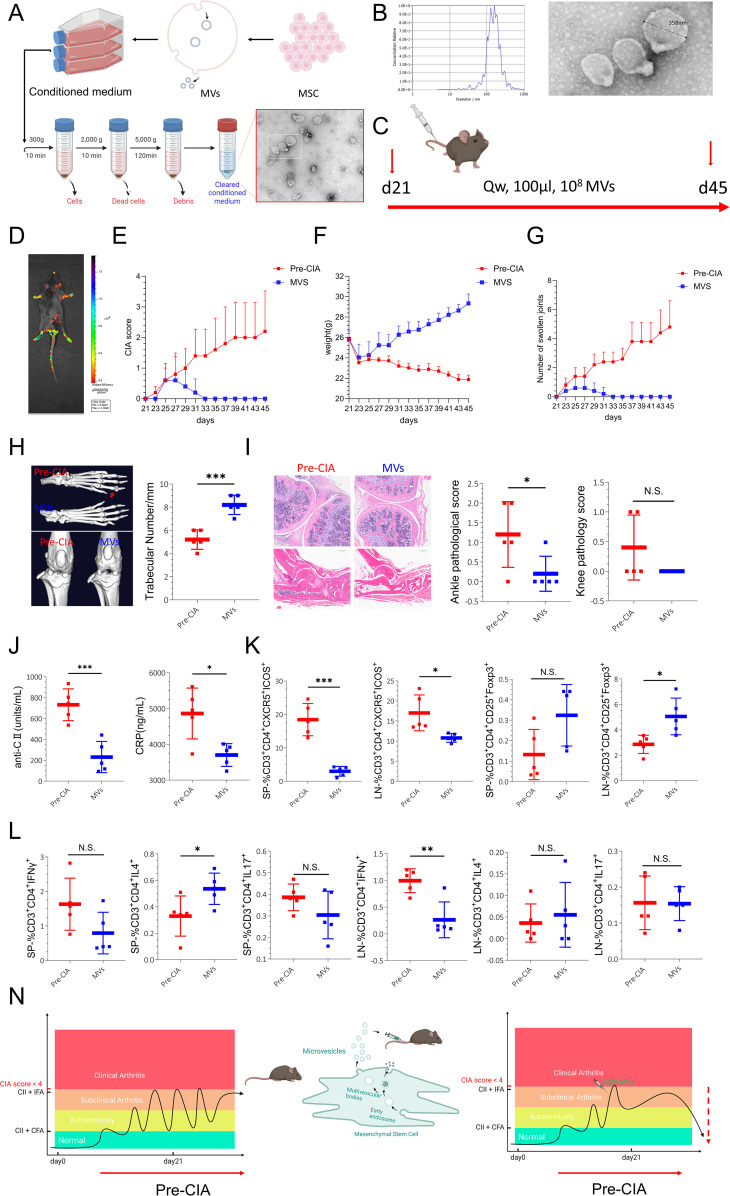
Treatment with MSC-MVs Was Safe and Effective for Treating Pre-CIA. **(A)** Experimental protocol for isolating MVs using differential ultracentrifugation. **(B)** Identification of MVs: The obtained MVs were detected for particle size; 95.00% of MVs were between 100 nm and 1000 nm. Transmission electron microscopy showing the cystic structure of MVs. **(C)** Experimental design. **(D)** MVs (DiL-Red) were tracked with live fluorescence imaging. **(E)** Changes in arthritis scores of mice (days 21-45). **(F)** Changes in the weight of mice (days 21-45). **(G)** The incidence of arthritis (days 21-45). **(H)** CT 3D reconstruction of toe and knee joints and trabecular number (TN./mm). **(I)** Representative H&E staining (scale bar, 500µm) and pathological scoring of mouse joints (scoring criteria: synovial hyperplasia (+1) point, cartilage destruction (+1) point, joint severe damage (+1) point). **(J)** Serum levels of anti-CII and CRP in mice. **(K-L)** Flow cytometry for measuring the proportion of Tfh, Treg, Th1, Th2, and Th17 cells in the inguinal lymph nodes and spleens of mice. Data are expressed as mean ± SD. (N) Schematic representation of the experiment in which Pre-CIA was modeled and treated with MSC-MVs to return Pre-CIA to normal. Data were analyzed using one-way ANOVA followed by Tukey’s *post-hoc* test. Two-tailed p-values <0.05 were considered significant. *P<0.05, **P<0.01, ***P<0.001, ****P<0.0001, N.S. not significant.

**Figure 8:**
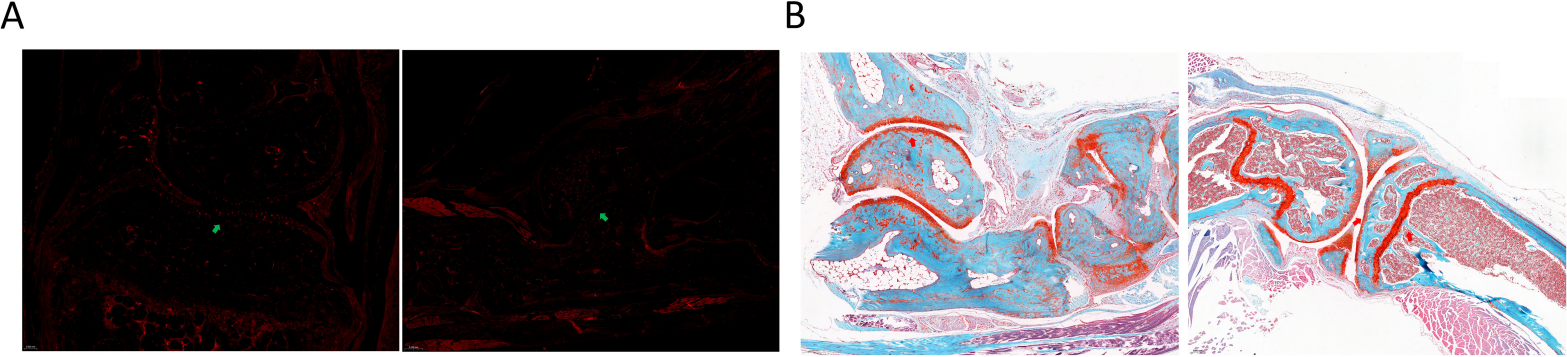
**(A)** Distribution of MVs in the joint. Fluorescence imaging of frozen sections shows DiI-labeled MVs (red) localized at the articular surface. Note: Erythrocytes also exhibit red autofluorescence but are morphologically distinct. **(B)** Histopathological assessment. Safranin O-Fast Green staining shows joint destruction. Red arrows indicate areas of cartilage damage and proteoglycan loss.

MVs were extracted using gradient centrifugation (**Figure 7A**). Nanoparticle tracking analysis confirmed that the sizes of MVs were between 100 nm and 1000 nm. Transmission electron microscopy showed vesicular structures within the same range of size (**Figure 7B**). Starting from day 21, MVs were injected intravenously (**Figure 7C**). *In vivo*, fluorescence imaging revealed MV accumulation near the joints (**Figures 7D**, **8A**). CIA scores, body weight, and joint swelling were measured every other day during treatment. CIA scores were reduced to zero in MV-treated mice, with gradual weight gain and no joint swelling, and there were significant differences between MV-treated mice and the Pre-CIA group (**Figures 7E-G**). Post-treatment CT scans and 3D reconstructions demonstrated that compared to the Pre-CIA group, treatment with MV significantly (P < 0.001) stabilized trabeculae, with no significant joint surface damage (**Figure 7H**). H&E staining of decalcified joint sections showed no significant synovial hyperplasia or cartilage changes in MV-treated joints, and pathological scores were significantly different from those observed in the Pre-CIA group (**Figures 7I**, **8B**). ELISA indicated reduced levels of anti-CII and CRP (**Figure 7J**). Flow cytometry of inguinal lymph nodes and spleens showed decreased Tfh and Th1 proportions, increased Treg and Th2 proportions, and reduced Th17 proportions. These results suggest that treatment with MVs stabilized the systemic immune status in Pre-CIA mice (**Figures 7K, L**). This provided modeling and therapeutic possibilities for Pre-RA (**Figure 7N**).

## 4 Discussion

RA is a chronic inflammatory disease characterized by an abnormal immune response (33, 34). Genetic and environmental factors are the primary mechanisms leading to the misrecognition of self-antigens, resulting in the activation of T cells and B cells and the production of autoantibodies, such as rheumatoid factor (RF) and ACPAs (35, 36). CIA is a commonly used mouse model of RA that involves immunizing mice with CII to induce an immune response similar to RA, characterized by joint inflammation, synovial hyperplasia, and bone destruction. The CIA model replicates several pathological features of RA, including the production of autoantibodies (e.g., anti-CCP and anti-CII) (37, 38), activation of T and B cells (39–41), and the release of cytokines (e.g., TNF-α, IL-1, and IL-6). Helper T cells, particularly Th1 and Th17 cells, are significantly more abundant in patients with RA, whereas Th2 cells are less abundant in such patients, leading to a Th1/Th2 imbalance (42). Additionally, the number and function of Treg cells are impaired, reducing immune tolerance to self-antigens and resulting in a Th17/Treg imbalance (43). The abundance of Tfh cells increases in RA and their abnormal function is closely associated with autoantibody production and B cell-mediated inflammatory responses in this disease. The induction and progression of CIA are regulated by genetic (DBA/1J mice) (44) and immune factors, similar to the pathogenesis of RA.

Pre-RA may last from months to years, during which the gradual dysregulation of the immune system leads to synovial inflammation and articular damage, representing a critical period for the development of RA (45, 46). Studies on Pre-RA can help identify high-risk individuals and develop early intervention strategies, which are essential for preventing or decelerating disease progression. Moreover, investigating the immune and environmental factors in the preclinical phase can help unravel the pathogenesis of RA, thereby identifying new therapeutic targets and prevention strategies.

By modifying the CIA model protocol, we developed a novel mouse model with Pre-RA characteristics, known as Pre-CIA. Pre-CIA mice exhibited an immunoactive state with symptoms of mild arthritis, mimicking the pathological characteristics of RA in the preclinical phase. In the CIA model, mouse CD4+ T cells were activated by subcutaneous injection of CFA and CII, inducing an inflammatory response (47, 48). We found that mice exhibited immune dysregulation after the first injection of CFA and CII, and the inflammatory response after the second injection led to the development of an arthritis model. This gradual development of arthritis closely resembles the clinical features of RA. Although the Pre-CIA model was highly similar to Pre-RA in mimicking its early immunopathological features, there remain certain limitations. Firstly, the model strongly relies on specific genetic backgrounds. It can be successfully induced only in genetically susceptible strains, which restricts its representation of human genetic diversity. Secondly, the pathological mechanisms of the Pre-CIA model predominantly originate from autoimmune responses against CII, whereas the etiology of human Pre-RA is more complex and may rely on several antigens. Finally, although the Pre-CIA model recapitulates many pathological features of Pre-RA, they have notable differences in certain aspects that should be considered.

We established criteria for defining the Pre-CIA model. To be qualified as Pre-CIA, the mouse model must meet the following conditions: (1) the CIA score should be ≤3 points, and/or micro-CT should show erosions in ≤3 joints; (2) the joints may not show arthritis, but immune activation or collagen loss should be evident in the articular cartilage, evidenced by a Safranin O-Fast Green staining score of >1; (3) the Pre-CIA model can be induced into the CIA model by LPS, resulting in severe arthritis. This “second-hit” (49) mechanism may more accurately reflect RA and can be more suitable for studying its pathogenesis. Currently, RA research mainly focuses on the post-diagnosis stage due to difficulties in obtaining early-stage samples. Therefore, the Pre-CIA model provides an accessible and early intervention model for RA research.

MSCs possess immunomodulatory, anti-inflammatory, and regenerative properties, making them ideal candidates for treating RA. Numerous preclinical and clinical studies have shown their potential to improve the symptoms and function of those with RA without severe adverse reactions (50, 51). However, the complexity of cell therapy limits the development of MSCs, and MSC-based paracrine research is emerging as an alternative treatment. MSC-MVs have shown immunomodulatory and reparative effects in the CIA model. We found that intravenous injection of MSC- MVs can regulate the immune response and restore the immune balance in Pre-CIA mice. MSC-MVs are small membrane-bound vesicles, 100–1000 nm in diameter, containing various bioactive molecules, such as proteins, RNA, and lipids, facilitating intercellular communication (52, 53). MSC-MVs play a critical role in immunoregulation by delivering anti-inflammatory factors, such as IL-10, TGF-β, and HLA-G, inhibiting inflammatory responses and excessive immune cell activation, thereby alleviating immune-mediated diseases, such as RA (60). MVs also promote the generation of regulatory T cells (Tregs), which maintain immune tolerance. Moreover, MSC-MVs carry specific miRNAs (e.g., miR-150 and miR-223) (54, 55) that regulate gene expression, inhibit the proliferation of synovial cells, and downregulate the production of pro-inflammatory cytokines, thereby mitigating synovial inflammation (56, 57).

In our study, after the injection of MVs, Pre-CIA joints returned to normal, and the immune state was stabilized, consistent with the results of previous studies. MSC-MVs did not show any significant side effects in the treatment process. In this study, we focused on investigating whether MSC-MVs can prevent the progression of Pre-CIA to CIA. However, we did not comprehensively explore the specific molecular targets.

Cohort studies have shown that early treatment of RA can delay disease progression and significantly improve patients’ prognosis. Intervention in the preclinical phase is an increasing trend in healthcare. Taking appropriate measures against causative or risk factors before disease development can prevent active disease (11). The concept of preventing the disease before its onset will be increasingly recognized in the future, fulfilling the notion of “preventive treatment”; thus, patients can achieve the utmost benefit (58, 59). However, Pre-RA represents a nearly healthy population, and clinical trials for them are not ethical. Therefore, establishing a reasonable model and improving the model validation and disease activity evaluation system is crucial for studying the pathogenesis, treatment, and prevention of Pre-RA. Thus, it is vital to develop drugs that can be used in healthy individuals to prevent arthritis. MSC-derived MVs are promising for the prevention and treatment of Pre-RA.

## 5 Conclusions

By adjusting the dose of CIA-inducing agents, we established a mouse model characterized by immune dysregulation without the immediate onset of arthritis, known as the Pre-CIA model. This model reflects the clinical features of Pre-RA in patients, representing immune disturbances that can progress to CIA after stimulation with LPS. Using this model, we investigated the role of MSC-MVs in Pre-CIA and found that MSC-MVs can effectively prevent the progression of Pre-CIA to CIA. This study holds significant clinical implications for the prevention, treatment, and prognosis of RA.

## Data Availability

The original contributions presented in the study are included in the article/supplementary material. Further inquiries can be directed to the corresponding authors.
